# Impact of sleep health on self-perceived health status

**DOI:** 10.1038/s41598-019-43873-5

**Published:** 2019-05-13

**Authors:** Mireia Dalmases, Ivan Benítez, Esther Sapiña-Beltran, Oriol Garcia-Codina, Antonia Medina-Bustos, Joan Escarrabill, Esteve Saltó, Daniel J. Buysse, Rosa E. Plana, Manuel Sánchez-de-la-Torre, Ferran Barbé, Jordi de Batlle

**Affiliations:** 1 Group of Translational Research in Respiratory Medicine, Hospital Universitari Arnau de Vilanova and Santa Maria, IRBLleida, Lleida, Spain; 2 Subdirecció General de Planificació Sanitària i Professional, Catalan Health Department, Barcelona, Spain; 3 Master Plan for Respiratory Diseases (PDMAR), Catalan Health Department, Barcelona, Spain; 4 Center for Sleep and Circadian Science, University of Pittsburgh, Pittsburgh, PA USA; 5 Centro de Investigación Biomédica en Red de Enfermedades Respiratorias (CIBERES), Madrid, Spain

**Keywords:** Epidemiology, Risk factors

## Abstract

Although sleep habits have long been recognized as a promoter of health, the World Health Organization 2014 report on non-communicable diseases (NCDs) only listed smoking, alcohol intake, diet and physical activity (PA) as key modifiable risk factors that could enhance health and prevent NCDs. Cross-sectional data on 4385 surveys from the 2015 Catalan Health Survey, representative of the 2015 non-institutionalized Catalan population over age 14, were used to assess and compare the independent associations of low PA (International Physical Activity Questionnaire (IPAQ): low activity); poor diet (PREvención con DIeta MEDiterránea questionnaire (PREDIMED): low-adherent); poor sleep health (Satisfaction, Alertness, Timing, Efficiency and Duration scale (SATED): <8); smoking status; and, alcohol intake (high-risk drinker based on standard drink units) with having a poor self-perceived health status. Logistic regression models adjusted by age, gender, education level and number of comorbidities showed that poor sleep health had the strongest independent association with poor self-perceived health status (OR = 1.70; 95%CI: 1.37–2.12), followed by poor diet (OR = 1.37; 95%CI: 1.10–1.72) and low PA (OR = 1.31; 95%CI: 1.01–1.69). This suggests that sleep habits should be included among the important modifiable health risk factors and be considered a key component of a healthy lifestyle.

## Introduction

Having a healthy lifestyle is key to enjoying good health and avoiding non-communicable diseases (NCDs). In this sense, the World Health Organization (WHO) 2014 report on NCDs cited reduction in alcohol, salt/sodium and tobacco use; increased physical activity (PA); and, halting the rise of the hypertension, diabetes and obesity as some of the global targets for the control of NCDs^1^. Similarly, Ezzati and Riboli listed smoking, alcohol consumption, excess weight and obesity, diet and nutrition, and PA in their summary of behavioural and dietary risk factors for non-communicable diseases published in 2013^2^. Interestingly neither the 280-page long WHO report nor the highly cited article referred to sleep health or sleep habits as a potentially modifiable factor that could enhance health and prevent NCDs.

The interest in healthy sleep as a promoter of health has been documented since the origins of medicine, through the Middle Ages and up to the present. Short sleep duration has specifically been associated with hypertension^3,4^, cerebrovascular diseases^5^, coronary heart diseases^6,7^, cancer^5,8^, obesity^9^, diabetes^10^, and all-cause mortality^11,12^. Similarly, long sleep duration has been associated with adverse health outcomes^10,11,13^. Growing evidence suggests that sleep habits beyond sleep duration, such as changes in sleep timing due to shift work^14,15^ or even subjective sleep quality^16,17^, could be associated with NCDs, quality of life and overall health status.

In this context, it is reasonable to ask whether sleep health is a neglected pillar of health. Using data from the 2015 Catalan Health Survey (ESCA)^18^, the authors aimed to assess and compare the relative weights of diet, PA, smoking, alcohol consumption and sleep in relation to self-perceived health status.

## Methods

### Design, population and data collection

This is an observational cross-sectional study based on the ESCA 2015 survey^18^. The study population and sampling methodology have been described elsewhere^19^. Briefly, a multistage probability sampling method was used to obtain a representative sample of the non-institutionalized population in Catalonia in 2015. Up to 5598 surveys were obtained in two waves using computer-assisted interviews. Full details on the interviewing methodology have been described elsewhere^19^. For the purpose of this manuscript analyses a total of 4385 surveys corresponding to people above 14 years-old and not working night shift were considered.

The survey consisted on almost 500 questions including sociodemographic variables; health status & health-related quality of life; chronic diseases; unintentional injuries; pharmacological treatment; daily life limitations and disability; preventive practices; social support; mental wellbeing; dietary habits; PA and mobility; tobacco; alcohol; cannabis; use of healthcare resources during last 15 days and last year; material deprivation; and, sleep health. A full description of the ESCA 2015 questionnaire can be found elsewhere^19^ and the complete survey, in Catalan or Spanish, is publicly available from the Catalan Government web site (http://salutweb.gencat.cat/ca/el_departament/estadistiques_sanitaries/enquestes/esca/ [Accessed: August 20^th^, 2018]).

Sleep health was assessed using the SATED scale (Satisfaction, Alertness, Timing, Efficiency and Duration)^20^. Briefly, SATED is the result of a comprehensive review of the literature on sleep dimensions and their association with specific health outcomes in an attempt to create a tool capable of quantifying sleep health. SATED assesses 5 dimensions of sleep health by means of 5 questions: sleep Satisfaction (“Are you satisfied with your sleep?”); Alertness during waking hours (“Do you stay awake all day without dozing?”); Timing of sleep (“Are you asleep, or trying to sleep, between 2:00 a.m. and 4:00 a.m.?”); sleep Efficiency (“Do you spend less than 30 minutes awake at night? This includes the time it takes to fall asleep and awakenings from sleep”); and sleep Duration (“Do you sleep between 6 and 8 hours per day?”). Respondents indicate the frequency with which they experience or engage in each dimension, with answers ranging from 0 to 2 points (0 = “never” or “very rarely”; 1 = “sometimes”; 2 = “often” or “always”). Items on the SATED scale can be totalled to produce a single summary score, ranging from 0 (very poor sleep health) to 10 (excellent sleep health). For the purpose of this study having a SATED score <8 was considered as poor sleep health; this threshold corresponds to the median for this population as published elsewhere^19^.

The *PREvención con DIeta MEDiterránea* (PREDIMED) questionnaire^21,22^, measuring adherence to Mediterranean diet, was used as a measure of the healthiness of participants’ diet. Any score below 9 out of 14 questions was considered as a poor adherence to Mediterranean diet and thus poor diet, whereas scores of 9 or more were considered as a healthy diet. PA was measured using the International Physical Activity Questionnaire (IPAQ)^23,24^, that classifies subjects’ PA in low, moderate or vigorous. For the purpose of the analyses, we considered moderate and vigorous PA as a healthy PA and low PA as a poor PA. Smoking was assessed by a question on smoking status (never, former, current). Finally, an estimation of each subject’s risk of abusing alcohol was defined based on consumed standard drink units. Any weekly alcohol intake up to 28 standard drinks (“standard drinks” = 10 grams of pure alcohol) for men and 17 for women in the last 12 months was considered as low risk. Intake above these levels was considered high risk. Subjects without any alcohol intake in the last 12 months were considered as non-drinkers^25^.

Self-rated health status was assessed with the question: “In general, how would you rate your health” with the possible choices being “excellent”, “very good”, “good”, “fair”, or “poor”. For the current analyses, excellent, very good and good ratings were considered as good self-rated health status while fair and poor as a poor self-rated health status.

The ESCA survey is an official statistic of the Catalan Government. It was approved by the Consultants’ Committee of Confidential Information Management (CATIC) from the Catalan Health Department. ESCA was conducted in accordance to the Catalan and Spanish regulatory framework, in agreement with the year 2000 revision of the Helsinki Declaration. All participants in the ESCA survey were adequately informed and provided consent to participate. Data analysed in this study are included in this published article (Supplementary Information Files).

### Statistical analysis

Appropriate weighting adjustment was applied to achieve representative frequencies, as less populated territories were oversampled. Continuous variables were summarized as the mean (standard deviation) and categorical variables as percentages. Diet, PA, smoking, alcohol consumption and sleep health were assessed, individually and jointly, as determinants of poor self-rated health using a survey-weighted logistic model^26^ adjusted for age, gender, education level and number of comorbidities. A modelling of the effect of the number of risk categories on self-perceived health status was used to assess the additive effect of having multiple poor health behaviours. The conferred risks were estimated using odds ratios (OR) and 95% confidence intervals (CI). Differences in the classification accuracy of the models were assessed by comparing the area under the receiver operating characteristic curve (AUC). Goodness of fit was assessed using the Hosmer-Lemeshow calibration test. R statistical software, version 3.4.1, was used for all the analyses. All tests were two tailed, and p-values < 0.05 were considered statistically significant.

## Results

4385 surveys representative of the Catalan population were included in the analyses. The main sociodemographic, life-style and health-related characteristics of the population are shown in Table 1. Briefly, 49% of the sample were men, mean age (SD) was 47 (19) years, 58% were never smokers, 34% non-drinkers, 51% had a high adherence to Mediterranean diet and 15% reported vigorous PA. Regarding sleep health, the population had a SATED score of 7.91 (2.17), with a 67% of subjects reporting good sleep health (SATED ≥ 8). Finally, 81.7% of subjects reported themselves as having good self-rated health.

**Table 1:** Main characteristics of the population.

**Sociodemographic characteristics**	
Male gender	49%
Age (years)	47 (19)
Education level	
Primary	24%
Secondary	55%
University	21%
**Lifestyle habits**	
Tobacco use	
Current smoker	25%
Former smoker	17%
Never smoker	58%
Alcohol	
Non-drinker	34%
Drinker (low risk)	62%
Drinker (high risk)	4%
Diet (PREDIMED)	
Low adherence to Mediterranean diet	49%
High adherence to Mediterranean diet	51%
Physical activity (IPAQ)	
Low	26%
Moderate	59%
Vigorous	15%
Sleep health (SATED)	
Poor (SATED < 8)	33%
Good (SATED ≥ 8)	67%
**Health status**	
At least one chronic disease	72%
Good self-rated health status	82%

Proportion or mean (SD), as appropriate. PREDIMED: *PREvención con DIeta MEDiterránea* questionnaire; IPAQ: International Physical Activity Questionnaire; SATED: sleep Satisfaction Alertness Timing Efficiency and Duration scale.

**Figure 1:**
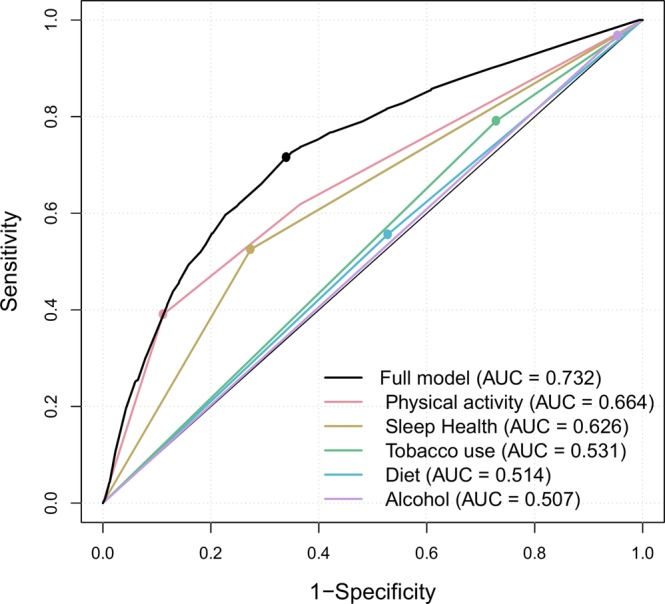
Receiver operating characteristic curves for the associations between life-style habits and self-rated health status. Logistic regression model. AUC: area under the curve.

Table 2 shows logistic regression models examining the associations between tobacco use, alcohol consumption, diet, PA and sleep health and poor self-rated health status, adjusted for age, gender and number of comorbidities. Having poor sleep health showed the strongest independent association with a poor self-perceived health status (OR = 1.72; p < 0.001), followed by low adherence to Mediterranean diet (OR = 1.41; p < 0.001) and being a current smoker (OR = 1.38; p = 0.01). The predictive capacity of each of these models according to Receiver operating characteristic (ROC) curves is compared in Fig. 1, and shows that PA (AUC = 0.664) and sleep health were the life-style factors that best identified individuals with poor self-rated health status (AUC = 0.626).

**Table 2:** Logistic regression models examining the association between tobacco use, alcohol consumption, diet, physical activity and sleep health and poor self-rated health status.

	OR (95%CI)	p-value
Tobacco		
Non-smoker	Ref	
Smoker	1.38 (1.07–1.79)	0.01
Alcohol		
Low risk drinker	Ref	
High risk drinker	1.25 (0.71–2.20)	0.43
Diet		
High-moderate adherence to Mediterranean diet	Ref	
Low adherence to Mediterranean diet	1.41 (1.12–1.77)	<0.001
Physical activity		
Vigorous- Moderate	Ref	
Low	1.34 (1.03–1.73)	0.003
Sleep health		
Good (SATED ≥ 8)	Ref	
Poor (SATED < 8)	1.72 (1.39–2.13)	<0.001

Logistic regression models adjusted for age, gender, education level and number of chronic diseases. SATED: sleep Satisfaction Alertness Timing Efficiency and Duration scale.

Table 3 shows the adjusted associations between tobacco use, alcohol consumption, diet, PA and sleep health and poor self-rated health status, in a single model that also adjusts for age, sex, education level and number of comorbidities. In this model, poor sleep health was the factor most strongly associated with poor self-perceived health status (OR = 1.70; p < 0.001). The associations for both smoking status and alcohol intake were not statistically significant.

**Table 3:** Logistic regression model examining the mutually adjusted associations between tobacco use, alcohol consumption, diet, physical activity and sleep health and poor self-rated health status.

	OR (95%CI)	p-value
Gender: female	0.96 (0.77–1.20)	0.71
Age		
<45 years	Ref	
45–64 years	2.15 (1.63–2.83)	<0.001
65–74 years	1.71 (1.25–2.72)	0.02
≥75 years	2.65 (1.35–5.19)	<0.001
Number of chronic diseases	1.47 (1.41–1.54)	<0.001
Education level		
Primary	Ref	
Secondary	0.74 (0.57–0.95)	0.02
University	0.57 (0.39–0.82)	<0.001
Non-smoker	Ref	
Smoker	1.29 (1.00–1.68)	0.05
Non-drinker/low risk drinker	Ref	
High risk drinker	1.18 (0.66–2.12)	0.57
High/moderate adherence to Mediterranean diet	Ref	
Low adherence to Mediterranean diet	1.37 (1.10–1.72)	<0.01
Vigorous/Moderate physical activity	Ref	
Low physical activity	1.31 (1.01–1.69)	0.04
Good sleep health (SATED ≥ 8)	Ref	
Poor sleep health (SATED < 8)	1.70 (1.37–2.12)	<0.001

A single logistic regression model adjusted for age, gender, education level, number of chronic diseases and all considered modifiable risk factors at the same time. SATED: sleep Satisfaction Alertness Timing Efficiency and Duration scale.

Finally, a model assessing the additive effect of having multiple poor health behaviours is shown in Table 4. Having multiple poor health behaviours was associated to increased odds of poor self-rated health. This relation was dose-dependent, demonstrating an additive effect of poor health behaviours.

**Table 4:** Adjusted logistic regression model examining the additive effect of having multiple poor health behaviours on poor self-rated health status.

	OR (95%CI)	p-value
Absence of health risk behaviours	Ref	
1 Health risk behaviour	1.27 (0.94–1.73)	0.13
2 Health risk behaviours	1.57 (1.13–2.18)	0.01
3 Health risk behaviours	2.66 (1.80–3.92)	<0.001
4 or 5 Health risk behaviours	5.18 (2.91–9.24)	<0.001

Model adjusted for age, gender, education level and number of chronic diseases.

## Discussion

In this study, including data from a representative population sample of 4385 individuals, we assessed and compared the relative weights of diet, PA, smoking, alcohol consumption and sleep health in relation to self-perceived health status. Poor sleep health showed the strongest independent association with a poor self-perceived health status. This association held even when adjusting for the number of comorbidities and when all studied lifestyle factors were simultaneously adjusted.

It is well-known that sleep duration, either being too short or too long, is related to a poor health status at all ages^27–31^. Additionally, although less evidence is available, sleep quality has also been related to health status, showing stronger relations than sleep duration in most studies^32,33^. However, few studies up to date have tried to assess simultaneously the impact of sleep together with other key lifestyle factors in relation to health status, and only two of them included all the key modifiable risk factors identified by the WHO: smoking, alcohol intake, diet and PA^34,35^. For instance, Bayán-Bravo *et al*. prospectively assessed the impact of traditional (non-smoking, being very or moderately active and having healthy diet) and non-traditional (sleeping 7–8 h/d, being seated <8 h/d, and seeing friends every day) health behaviours in relation to health-related quality of life (HRQL) in a cohort of 2,388 subjects aged ≥60. PA, adequate sleep duration and sitting less, were associated with better HRQL in the short-term follow-up (2.5 years), whereas in the long term (8.5 years) only PA showed a significant association with HRQL^34^. However, this study assumed that health behaviours were stable over time, and did not assess dimensions of sleep health other than duration. Both limitations are relevant to the changes in sleep patterns usually seen with ageing: an increase in sleep duration combined with a decrease in sleep quality^19^. Duncan *et al*. assessed the cross-sectional associations of smoking, physical activity, diet, sitting time, sleep duration and sleep quality on self-perceived health among 10,478 individuals. Poor sleep quality, followed by low PA, had the strongest associations with self-perceived health^35^. Finally, several studies have assessed the combined effects of sleep duration and/or quality and other behavioural risk factors in relation to different health outcomes, all of them identifying significant relationships between sleep health and the studied health outcomes^36–40^.

The results of this study suggest that having healthy sleep, and thus being satisfied with the way we sleep, is a key factor in the self-perception of health. Individuals describing their sleep health as poor are less likely to report good self-perceived health than individuals engaging in other unhealthy habits such as tobacco smoking or alcohol consumption. This suggests that the link between sleeping well and feeling well is, if anything, tighter than the link with other well-known unhealthy habits. However, these associations do not necessarily imply that that poor sleep habits are the strongest modifiable health risk factor. People’s perceptions of sleep could lead to ratings of poor perceived health via reverse causality: “I perceive that I am not healthy because I don’t feel rested when I get up in the morning” or “I am struggling to get asleep every night thus my health cannot be good”. Therefore, the current results should not be interpreted as a proof of sleep health being the strongest modifiable risk factor for overall health status, but as an indication that sleep health may have been neglected when considering modifiable risk factors related to health.

From a public health perspective, the current study increases the evidence relating sleep health with overall health status. The promotion of healthy sleep habits could, therefore, be considered as a potential strategy to promote not only the overall health status of a given population, but also the self-perception of being healthy. This study also shows that having multiple poor health behaviours is associated to increased odds of poor self-rated health. Therefore, the promotion of healthier sleep habits could have a synergistic effect with other health promotion activities^35,41^.

The current study has several strengths, including: (i) a large sample size, selected to be representative of the Catalan population; (ii) comprehensive measures of behavioural risk factors; and, (iii) a detailed assessment of sleep health including multiple dimensions. On the other hand, several limitations must be acknowledged: (i) the cross-sectional design did not allow establishment of the direction of the associations and, therefore, whether poor sleep health is a cause or a consequence of a poor self-perceived health status falls beyond the scope of the current analyses; (ii) the SATED scale has yet to be formally validated, although their five dimensions have been consistently associated with health outcomes and no other validated tool is currently available to measure sleep health; (iii) all behavioural risk factors were self-reported and thus potentially subject to some degree of reporting bias and/or social desirability bias; and, (iv) although the dichotomization of the behavioural risk factors allowed for a direct comparison of the magnitude of their effects, it also implied some degree of misclassification. This could be especially relevant in the case of former smokers who quitted because of medical conditions.

In conclusion, our study suggests that sleeping habits are amongst the strongest potentially modifiable risk factors associated with the self-perception of health, independent of age, gender and the number of comorbidities. Although poor sleep health can be a cause or a consequence of a poor self-perceived health status, these findings show that sleep habits should not be neglected when defining a healthy lifestyle.

## Supplementary information

Dataset 1
